# An Audit of the Use of Total Parenteral Nutrition: Are We Using It Correctly?

**DOI:** 10.7759/cureus.102333

**Published:** 2026-01-26

**Authors:** Anil Kumar, Meghana Taggarsi, Sajal Rai

**Affiliations:** 1 Colorectal and General Surgery, Stepping Hill Hospital, Stockport NHS Foundation Trust, Stockport, GBR; 2 Colorectal and General Surgery, Pilgrim Hospital, United Lincolnshire Teaching Hospitals NHS Trust, Boston, GBR; 3 Colorectal and General Surgery, George Eliot Hospital NHS Trust, Nuneaton, GBR

**Keywords:** bmi, dietician, malnutrition, nice guidelines, nutrition, re-feeding, total parenteral nutrition, tpn

## Abstract

Background

Total parenteral nutrition (TPN) provides essential nutritional support when the gastrointestinal (GI) tract is non-functional or inaccessible. Its use should be limited to situations where enteral feeding is not possible or contraindicated. Enteral nutrition remains the preferred option whenever feasible. Clinicians should ensure an informed discussion regarding the relative risks and benefits of parenteral versus enteral nutrition.

Objectives

To evaluate adherence to local and national guidelines in the use of TPN, including frequency of emergency TPN prescriptions, duration of therapy, and dietitian review of TPN regimens.

Methods

This retrospective audit was conducted over a three-month period from August to October 2019. All adult patients prescribed TPN under general surgery were included, while those managed in the intensive care unit were excluded. Data were collected on TPN indication, assessment of alternative feeding routes, body mass index (BMI)/weight changes, duration, prescription appropriateness, delivery route, dietitian review, and complications. Findings were benchmarked against the National Institute for Health and Care Excellence (NICE, 2017) and the National Confidential Enquiry into Patient Outcome and Death (NCEPOD, 2010) standards, and compared with three previous audit cycles (2013-2016). Data were analysed descriptively, with categorical variables expressed as counts and percentages. Differences across audit cycles were assessed using the chi-square or Fisher’s exact test, with p < 0.05 considered statistically significant.

Results

Sixteen patients met the inclusion criteria. Indication for TPN was documented for 100% of the patients. Thirteen (81.25%) of our patients had other modes of feeding excluded. The Malnutrition Universal Screening Tool (MUST) was used for all patients included in the study. Fifteen (93.75%) patients had no documentation of their weight/BMI at the end of the TPN regimen. Five (31.25%) patients were started on emergency TPN by an out-of-hours doctor. All the patients had their TPN prescribed adequately. None of our patients had any significant metabolic complications or re-feeding syndrome.

We compared the outcomes of this audit with the previous three cycles. There was improved documentation of the indication of TPN across four cycles. Statistically significant improvement was found in the dietitian review (p = 0.002) and correct TPN prescription (p < 0.001). Documentation of BMI/weight declined across four cycles (p = 0.008). The majority of TPN was dietitian-led, and the need for TPN was reviewed regularly.

Conclusion

This study highlights the importance of multidisciplinary team involvement to improve care in TPN delivery. All efforts should be taken to use the GI tract for nutrition provision if it is available for use. The findings of this audit may relate to local practices; however, the lessons learnt may be applicable to other healthcare systems seeking to review and improve governance and quality assurance in TPN service delivery.

## Introduction

Nutrition plays a very important role in the optimal recovery of a surgical patient. It is a fundamental, yet generally neglected aspect of surgical care. To understand the intricacy of nutrition in promoting postoperative recovery, a thorough insight into the surgical stress response is essential. Surgical trauma or stress leads to a hypermetabolic state characterised by hormonal, metabolic, immunological, and haematological changes [1]. This complex association between metabolic response and surgery paved the way for extensive research into perioperative replenishment of stress-induced nutritional deficiency. Decades of research have clearly, eloquently, and repeatedly demonstrated an association between inadequate nutritional status and poor surgical outcomes [2].

Malnutrition has been demonstrated to be an independent poor prognostic factor after surgery [3]. It is a complex pathology prevalent in approximately 30-50% of patients undergoing surgery and is associated with heightened morbidity, mortality, length of stay (LOS), and hospital cost. Since its inception, surgical nutrition has been a dynamic and evolving discipline with several decades of research to support perioperative nutritional requirements as a potent means of improving outcomes [4].

Route of nutrition delivery and total parenteral nutrition

To supplement the perioperative nutrition of a surgical patient, oral, enteral, and parenteral routes are commonly used. Total parenteral nutrition (TPN), invented by Dr. Stanley Dudrick, revolutionised the perioperative care [2]. It refers to the provision of required nutrients by the intravenous route wherein essential nutrients are administered in their elemental or pre-digested form, with proteins as amino acids, carbohydrates as dextrose, fat as lipid emulsion, and micronutrients like minerals, vitamins and electrolytes. According to the National Institute for Health and Care Excellence (NICE) guidelines, TPN should be considered in malnourished patients or in patients at risk of developing malnutrition in the perioperative period. TPN should only be considered if patients have an inadequate or unsafe oral and/or enteral intake and in those who have an inaccessible, non-functional, or perforated gastrointestinal (GI) tract [5].

TPN undoubtedly provides excellent nutritional supplementation. However, there are compelling risks associated with this route of supplementation, namely, metabolic derangements like hyper/hypoglycaemia, azotaemia, hypertriglyceridemia, electrolyte abnormalities, cholestasis, fatty liver, bacterial translocation, sepsis, re-feeding syndrome, and complications related to TPN access like thrombosis, air embolism, catheter fracture/displacement, and pneumo/haemothorax [6].

National and international guidelines (European Society for Clinical Nutrition and Metabolism, ESPEN) have been established to support nutrition in patients for whom the enteral route is not feasible [7]. There has been an increase in the use of the parenteral route of feeding in recent times. The American Society for Parenteral and Enteral Nutrition (ASPEN) has suggested the need for frameworks to guide hospitals to maintain competencies for safe administration of TPN [8].

We conducted an audit at our centre primarily to assess whether TPN is being used in accordance with the NICE guidelines and the National Confidential Enquiry into Patient Outcome and Death (NCEPOD) 2010 report, "A Mixed Bag", and to assess prescriptions for "Emergency TPN" [9]. We also evaluated the duration of TPN, appropriateness of TPN prescription, and nutritional monitoring. This audit also aimed to assess the dietitian's review of the prescribed TPN regimens to ensure their accuracy.

## Materials and methods

Study design

This study was undertaken at Stepping Hill Hospital, Stockport NHS Foundation Trust, Stockport, United Kingdom (UK) from August 2019 to October 2019. The audit was registered with the local audit department. The audit methodology and outcome measures were consistent with previous audit cycles to allow valid comparison over time.

Inclusion and exclusion criteria

All patients admitted to the general surgery facility, aged 18 years and above, and prescribed TPN were included in the study, while patients aged less than 18 years and patients under the care of the high dependency unit (HDU)/intensive care unit (ICU) having TPN were excluded from the study.

Data collection and statistical analysis

Patient data were obtained from the aseptic department. Within the National Health Service (NHS), the aseptic unit is a specialist pharmacy service where medicines such as chemotherapy and parenteral nutrition are prepared in a sterile, controlled environment, ensuring safe, ready-to-use treatments while reducing contamination risk. Data were collated from inpatient notes, local electronic medical records, electronic prescribing and medicines administration (ePMA) records, and from the dietary department.

The primary objective of this audit was to evaluate compliance with national standards for the use of TPN in general surgical patients, as defined by NICE (2017) and the NCEPOD report, "A Mixed Bag" (2010) [9]. The secondary objectives were to assess key quality indicators of TPN practice, including utilisation of the Malnutrition Universal Screening Tool (MUST) to assess the nutritional status of the patients at admission [10]. The other key quality indicators assessed included documentation of the clinical indication for TPN, confirmation that oral or enteral feeding routes had been appropriately considered and excluded, accuracy and appropriateness of TPN prescription, involvement of dietitians in initiation and review, frequency of out-of-hours ("emergency") TPN prescribing, duration of TPN administration, adequacy of nutritional monitoring (weight and BMI), route of TPN delivery, and the occurrence of TPN-related metabolic or line-associated complications. Data were collected retrospectively from clinical and electronic records to capture these predefined outcome measures in a systematic and reproducible manner.

This audit represents a re-audit, with findings systematically compared against three previous audit cycles conducted using the same study design and outcome measures. The initial audit was undertaken between December 2013 and February 2014, followed by a second cycle from October to December 2014, and a third cycle from October to December 2016.

Data were analysed using descriptive statistics, with categorical variables expressed as counts and percentages. Comparisons across audit cycles were made using the chi-square test to assess differences in proportions. Where expected frequencies were <5, Fisher’s exact test was applied. A p-value <0.05 was considered statistically significant.

## Results

**Figure 1:**
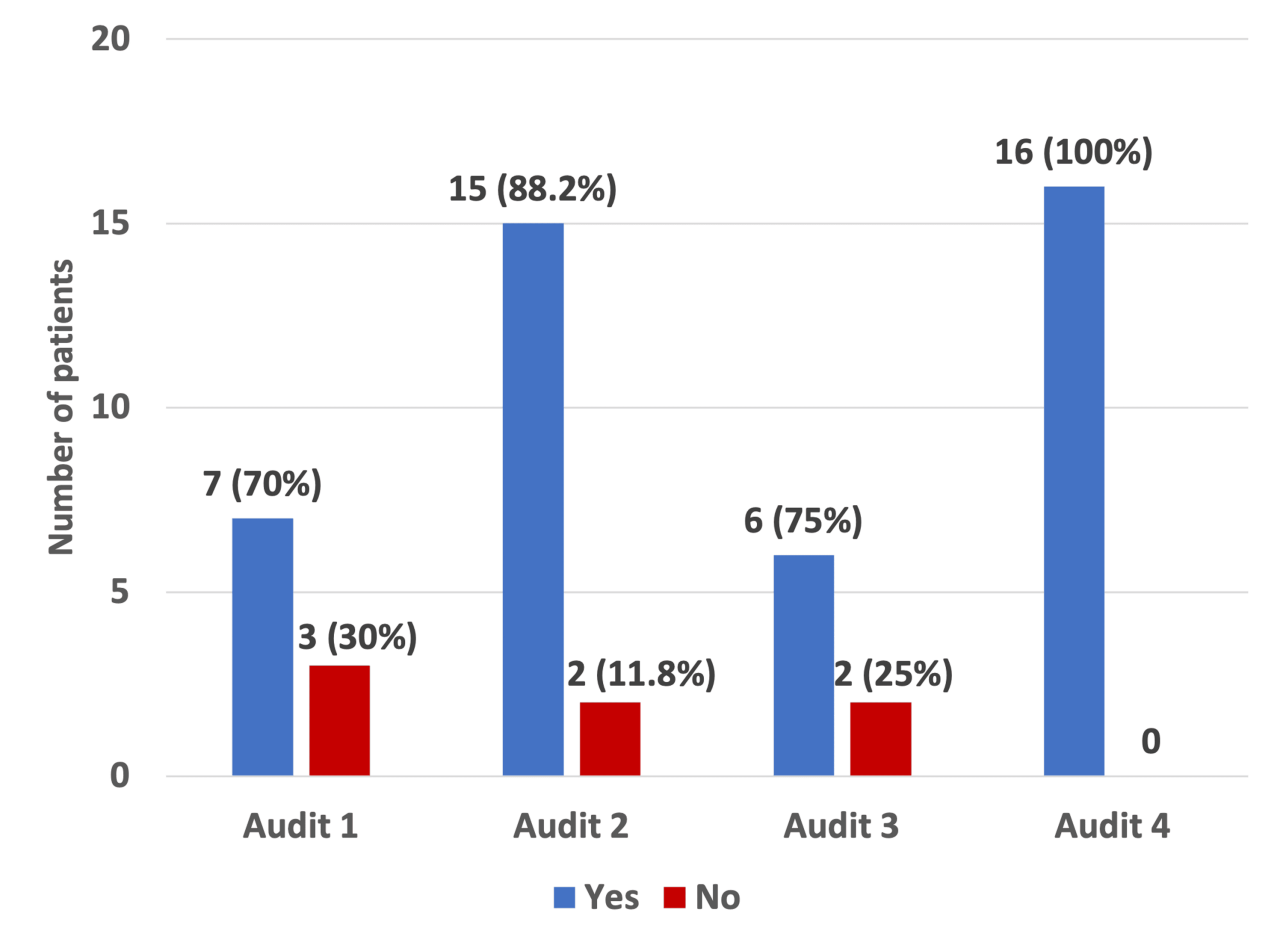
Documentation of indication of total parenteral nutrition in comparison with previous three audits.

In this audit cycle, a total of 26 patients who received TPN were identified, of which 16 patients met the study inclusion criteria. Data were collected using an Excel sheet (Microsoft Corporation, Redmond, WA). Indications for TPN were documented for all patients, amounting to 100% compliance with the guidelines (Figure 1). The index audit clearly demonstrates significant improvement in adherence to guidelines. The indications for TPN have been summarised in Table 1.

**Table 1:** Table summarising indication of TPN in our patients. TPN: total parenteral nutrition; NGT: nasogastric tube.

Indication for TPN	Number of patients
Oesophageal/bowel cancer	2
Oesophageal stricture/dysphagia	6
Gastric outlet/bowel obstruction	1
Postoperative Ileus/leak	3
Duodenal tumour	1
Keep pulling out NGT/nil by mouth	1
High output stoma/intestinal failure	1
Facial tumour removal	1

**Figure 2:**
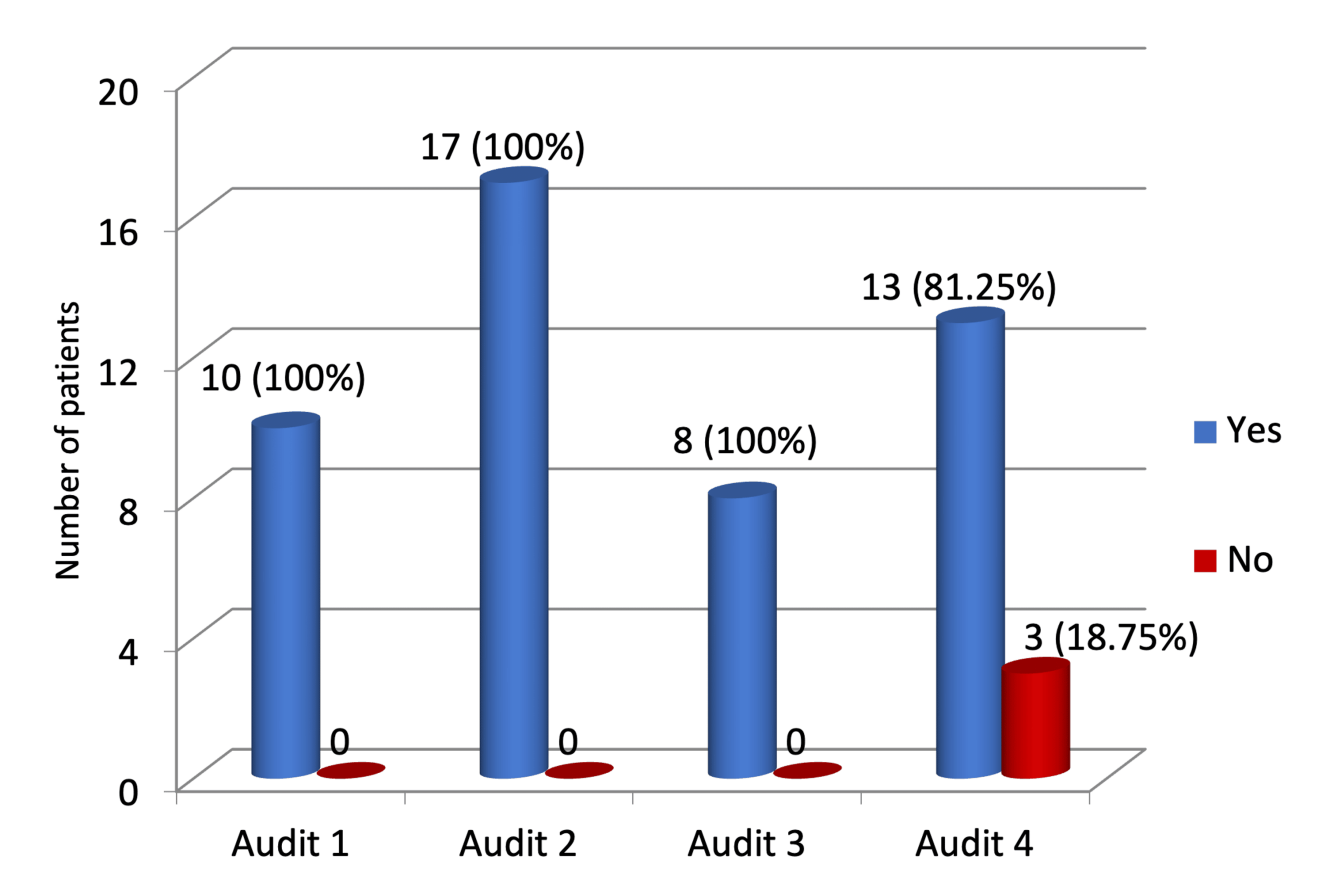
Exclusion of other methods of feeding.

A total of 13 (81.25%) of our patients had other modes of feeding excluded, while no documentation was found for three (18.75%) of the patients (Figure 2). All patients had their MUST screening done at the time of admission, which is in 100% compliance with the NICE guidelines.

**Figure 3:**
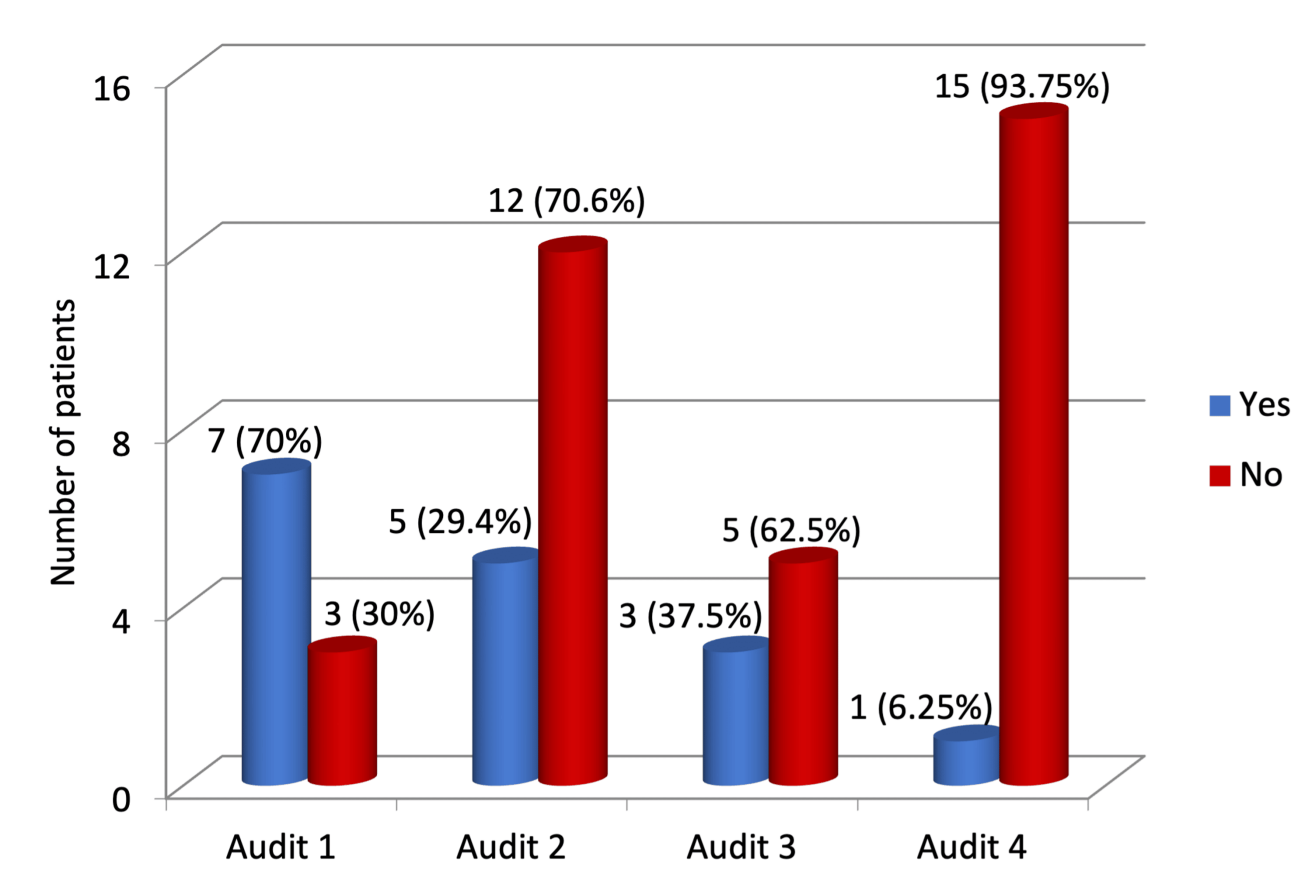
Documentation of BMI/weight at the start and end of total parenteral nutrition.

A total of 15 (93.75%) patients had no documentation of their weight/BMI at the beginning and end of the TPN regimen, while only one patient had adequate documentation of weight/BMI at the start and the end of the TPN regimen (Figure 3).

**Figure 4:**
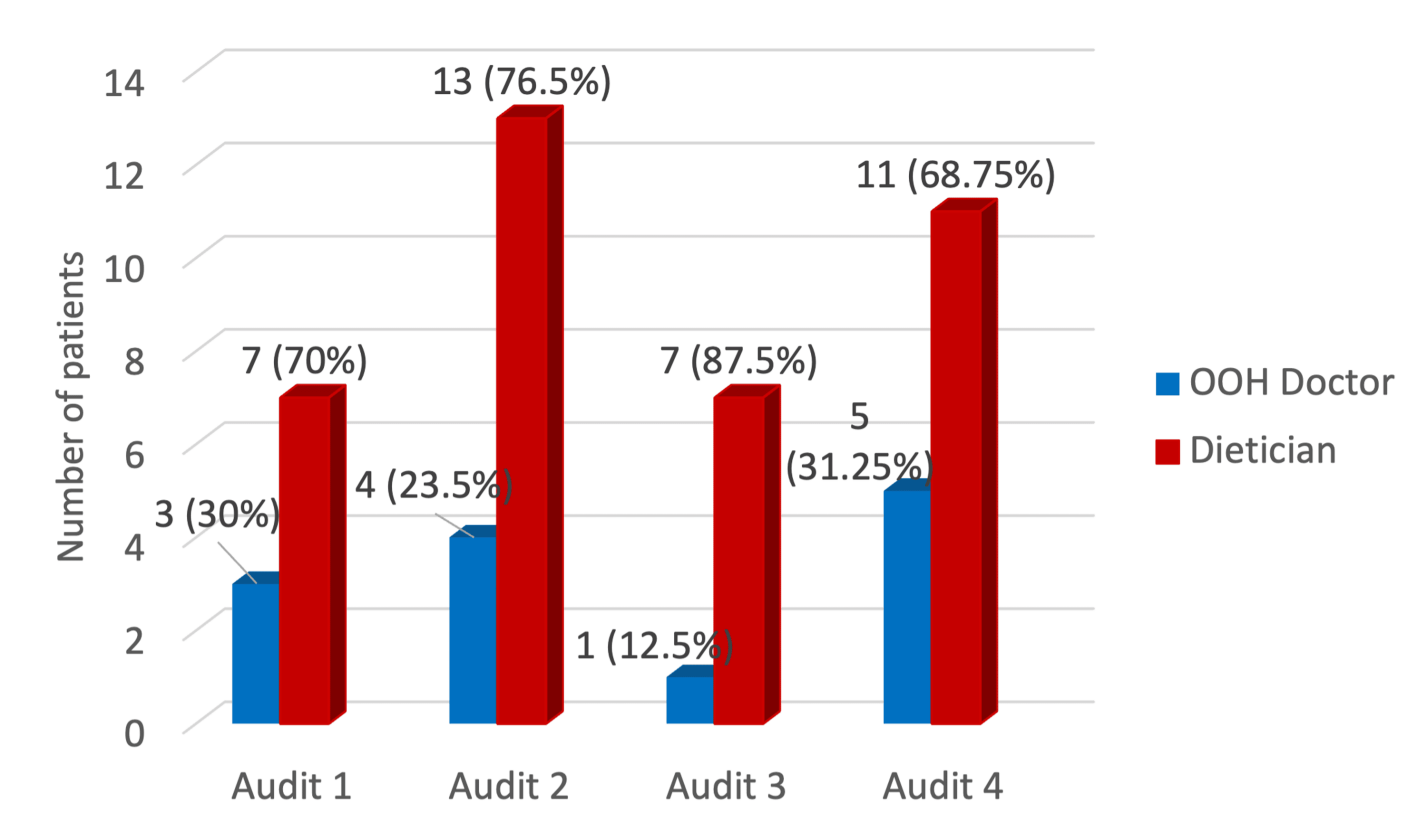
Who initiated the total parenteral nutrition? OOH: out-of-hours.

**Figure 5:**
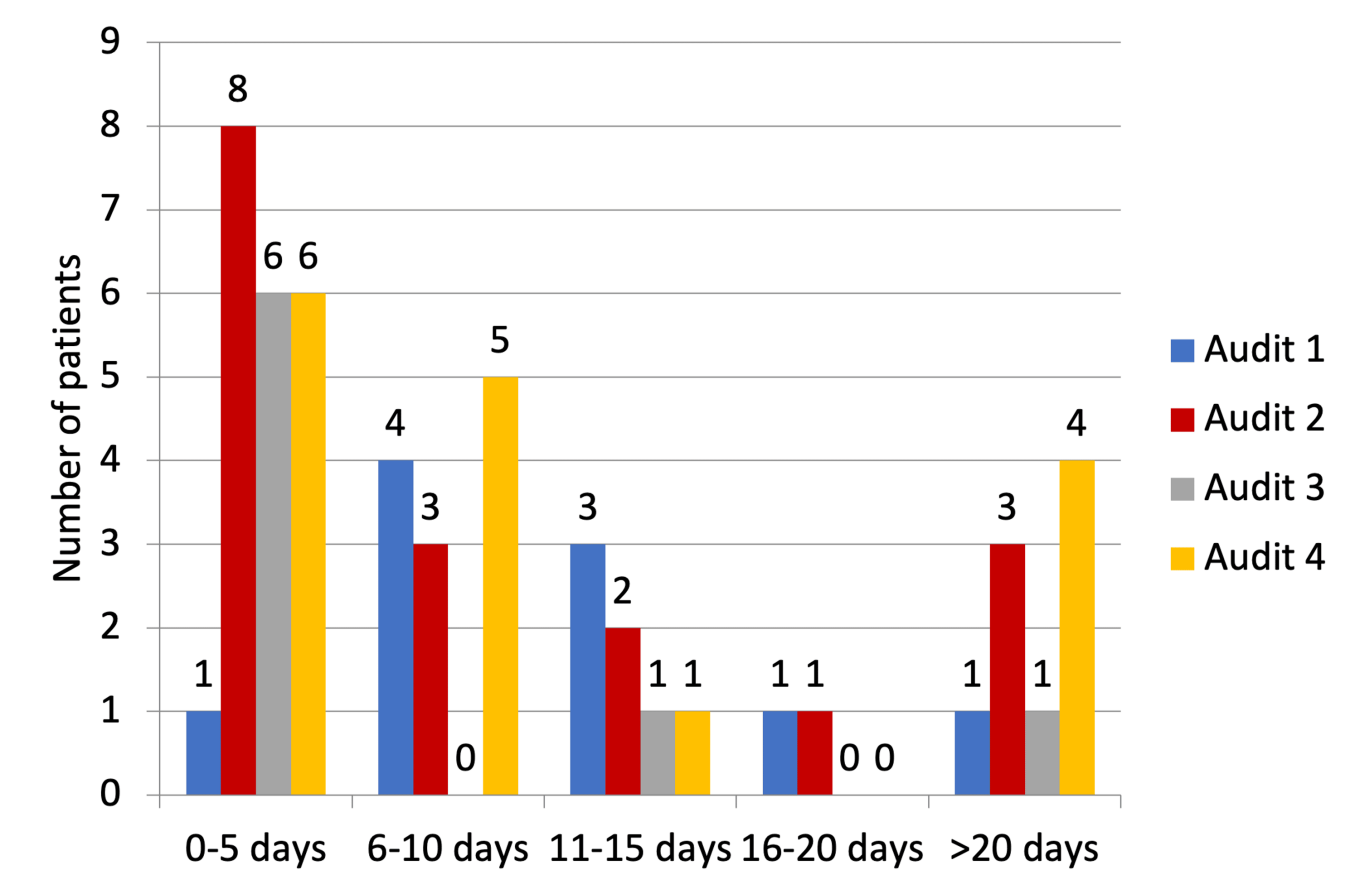
Duration of total parenteral nutrition given in days.

Five (31.25%) patients were started on emergency TPN by the out-of-hours (OOH) doctor, while in 11 (68.75%), it was the dietitian who initiated it (Figure 4). The duration of TPN was less than five days in six patients, 6-10 days in five patients, 11-15 days in one patient, and more than 20 days in four patients (Figure 5).

**Figure 6:**
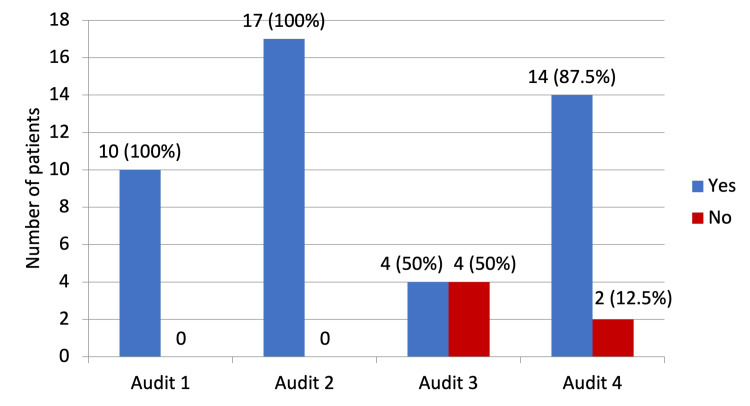
Documented serial dietitian review.

All the 16 patients had their TPN prescribed adequately, which is 100% complaint with the trust and NICE guidelines. Similarly, compliance was 100% in the first and second audit cycles; however, it was 50% in the third cycle. A total of 14 (87.5%) patients had a dietitian review, while no documentation of a dietitian review was found in two patients (Figure 6).

**Figure 7:**
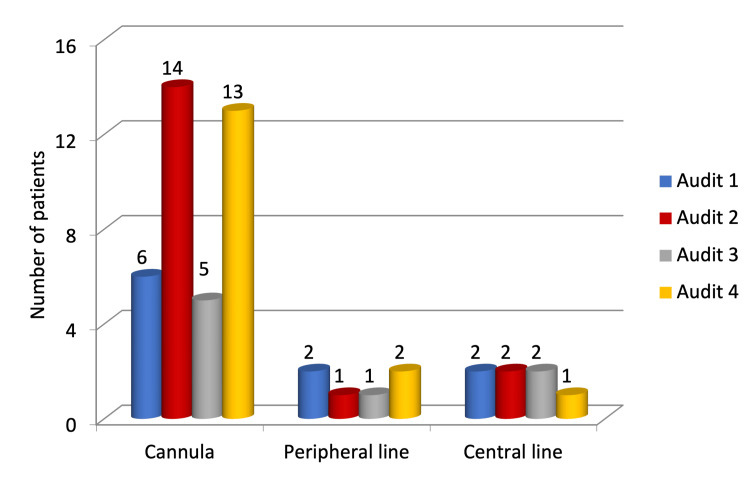
Route of delivery of total parenteral nutrition.

The majority of our patients (13, 81.25%) received TPN through an intravenous cannula, two patients were given TPN through the peripherally inserted central catheter (PICC), and the remaining one patient received TPN through a central venous catheter (CVC). None of our patients had any line-associated complications, metabolic disturbances, or re-feeding syndrome (Figure 7).

A total of 51 patients were included across the four audit cycles: 10 in Audit 1; 17 in Audit 2; 8 in Audit 3; and 16 in the current cycle.

Documentation of TPN indication improved steadily from 70% in Audit 1 to 100% in the present audit, although this change was not statistically significant (p = 0.127). Documentation confirming that other feeding methods were unsuitable remained high throughout, with a slight decline in the current cycle (81.3%; p = 0.073).

In contrast, BMI or weight documentation showed a marked decline from 70% in Audit 1 to just 6.25% in the present audit, a statistically significant finding (p = 0.008).

TPN initiation by a dietitian remained consistent across cycles (68.8%-87.5%), with the remainder initiated by OOH doctors; no significant variation was observed (p = 0.769).

Dietitian review, however, demonstrated marked improvement from 50% in Audit 3 to 87.5% in the current cycle, representing a statistically significant positive trend (p = 0.002) (Table 2).

**Table 2:** Statistical analysis of key parameters across four audit cycles. The chi-square test was used where expected cell frequencies were greater than or equal to five. Fischer's exact test was used when one or more expected count was less than five (notably for other feeding methods excluded). A p-value of <0.05 is considered statistically significant. TPN: total parenteral nutrition.

Variable	Audit 1 (n = 10)	Audit 2 (n = 17)	Audit 3 (n = 8)	Audit 4 (n = 16)	Test used	Degree of freedom (df)	Test value	P-value	Interpretation
Indication documented	7 (70%)	15 (88.2%)	6 (75%)	16 (100%)	Chi-square (X^2^)	3	X^2^=5.70	0.127	Not significant
Other feeding methods excluded	10 (100%)	17 (100%)	8 (100%)	13 (81.25%)	Fisher’s exact test	-	-	0.073	Not significant
BMI/weight documented	7 (70%)	5 (29.4%)	3 (37.5%)	1 (6.25%)	Chi-square (X^2^)	3	X^2^=11.79	0.008	Significant
TPN initiated by a dietitian	7 (70%)	13 (76.5%)	7 (87.5%)	11 (68.75%)	Chi-square (X^2^)	3	X^2^=1.13	0.769	Not significant
Dietitian review	10 (100%)	17 (100%)	4 (50%)	14 (87.5%)	Chi-square (X^2^)	3	X^2^=14.88	0.002	Significant
TPN correctly prescribed	10 (100%)	17 (100%)	4 (50%)	16 (100%)	Chi-square (X^2^)	3	X^2^=19.60	<0.001	Significant

Prescription of TPN showed an overall improvement across the four audit cycles (p < 0.001).

Overall, the audit shows a statistically significant improvement in correct TPN prescription and the rate of dietitian reviews. In contrast, the documentation of BMI and weight declined over time, a change that was also statistically significant. Other aspects, such as recording of indications, exclusion of alternative feeding methods, initiation of TPN, and its duration, remained largely unchanged across audit cycles.

## Discussion

In 2010, the UK NCEPOD published a report on parenteral nutrition, "A Mixed Bag". They aimed at investigating the care of inpatients receiving TPN in both adult and neonatal populations. The audit report focused mainly on indications for TPN, type of TPN, its prescribing, mode of delivery, insertion of catheter and line care, and complications. The audit investigated the care for 877 adults who were provided TPN, out of which only 19% were considered to represent good practice. This report suggested a dedicated attention to the in-hospital use of TPN to improve the quality of patient care [9]. We considered recommendations made by the NICE guidelines (2017) and the NCEPOD report "A Mixed Bag" and reviewed our results in these contexts [5,9].

At the outset, patient assessment should be robust to ensure that TPN is the appropriate nutritional intervention and that adequate TPN is administered. The documentation of the clinical purpose and the goal of TPN is of utmost importance [5,9]. In our audit, indication for TPN was documented for all patients, amounting to 100% compliance with the guidelines. Although our study did not investigate the treatment goal.

Assessment and exploration of the possibility of the use of the oral, enteral route, or combination should be assessed before prescribing parenteral nutrition. Guidelines state that TPN should be prescribed only after the possibility of enteral nutrition has been evaluated and deemed impractical or inappropriate [5,9]. In our audit, we found that there was a lack of documentation of the exclusion of other feeding methods in 18.75% of patients. In the national audit of 2010, one-third of patients received inadequate consideration of enteral nutrition, which is in conjunction with our audit [9].

A multicentre study done in 2017 that set up the Northern Nutrition Network (NNN) for the North of England recommends that a nutrition team be set up in each centre for assessment of nutrition, early establishment of need for TPN, as well as regular clinical and biochemical monitoring. The team should be multi-disciplinary and should include surgeon input [11].

Once the need for TPN is established, this should be rapidly actioned and started at the earliest opportunity. However, there is rarely, if ever, an indication to start adult TPN out of normal working hours. In our audit, the need for TPN was recognised early, and 68.75% of patients received TPN after dietitian input during working hours, while in 31.25% patients, emergency TPN was initiated by an OOH doctor. In comparison to our results, the national audit of 2010 and the audit performed by NNN reported 82% prescription during working hours [9,11].

It is recommended that regular documentation of clinical monitoring and TPN prescription should be mandatory. Monitoring should comprise daily weights (where possible) and documentation of the success of the TPN within the overall clinical picture [5,7,9]. In our audit, TPN was prescribed accurately and adequately in 100% of patients, which was in compliance with the guidelines. The majority of our patients (87.5%) had a dietitian review regularly, while in 12.5% of patients, there was no documentation of review by dietitians. A possible explanation could be the initiation of "Emergency" TPN by the OOH doctor, which would have been stopped prior to the dietitian review. The ESPEN guidelines updated in 2021 recommend that every centre should establish feeding protocols and standard operating procedures (SOPs) for the risks and feasibility associated with attaining the caloric requirement [12].

All patients in our audit had MUST screening and weight/BMI documentation at the time of admission, demonstrating complete adherence with the NICE guidelines. However, there was a significant lack of documentation of weight/BMI at the completion of the TPN regimen (93.75%). Such a high percentage of lack in documentation of weight/BMI could be confounded by the need for TPN for less than a week in the majority of our patients. Although NCEPOD national audit recommends daily weight measurements where possible, NICE guidelines advise daily weight/BMI measurements if there are concerns regarding fluid and electrolyte balance, but otherwise this can be abbreviated to weekly measurements.

Regular documentation of biochemical monitoring should be mandatory to ensure that avoidable metabolic complications never occur. The ESPEN guidelines stress regular monitoring of micro-nutrients and their replacement for safe use of TPN. The complications can range from dyselectrolytaemia to refeeding syndrome, which can be life-threatening. It can also cause hepatobiliary dysfunction [13]. All our patients had routine biochemical monitoring while on TPN. None of our patients had any significant metabolic complications or refeeding syndrome. The audit performed by NNN documented metabolic complications in 43%, out of which 13% were reported to be avoidable [11].

In our audit, a peripheral cannula was employed to administer TPN in 81.25% of patients, while a PICC line and CVC were used in one and three patients, respectively. There was appropriate documentation of verification of the position of the catheter tip in patients who needed a PICC line or CVC. This was found to be compliant with the NICE guidelines, the audit report of NNN, and the ESPEN guidelines [5,11,12]. Emphasis is on the importance of adhering to aseptic precautions while inserting and using the line for TPN. Line-related infections can be severe and life-threatening [11]. Line complications include thrombophlebitis, tissue necrosis due to the contents of TPN, blockage of the catheter, and central line-associated bloodstream infections (CLABSIs). In the United States, an audit suggested that CLABSIs are the cause of 14% of health-associated infections [14]. The risk of CLABSIs can range from 1.3% to 26.2% when CVC is used for parenteral nutrition [15]. The risk of complications can be further reduced by implementing catheter lock therapy, regularly reassessing the necessity of parenteral nutrition, prioritising enteral over parenteral support, maintaining proper glucose control, and avoiding overfeeding [15]. In our audit, most patients were given TPN for a short time, due to which the use of the peripheral line was more common compared to the PICC line and CVC. Our study reports no line-related infections.

The present data were compared with the data from previous audits. We observed a significant improvement in correct TPN prescription to 100% in the present audit (p < 0.001). Documentation of the indication for TPN also improved over time, reaching 100% in the current audit, although this did not reach statistical significance across audits (p = 0.127). TPN initiation remained predominantly dietitian-led, with no significant change in the proportion of emergency/out-of-hours TPN prescribed by doctors across audits (12.5-31.2%, p = 0.769), which remains higher than that recommended by NCEPOD’s "A Mixed Bag". Notably, 18.75% of patients in the present audit had no documentation of exclusion of alternative feeding methods, compared with 100% documentation in previous audits (p = 0.073). The most persistent deficiency identified was documentation of weight/BMI at the end of the TPN regimen, which was missing in 93.75% of patients, representing a statistically significant decline over the audit cycles (p = 0.008), consistent with trends observed in prior audits (30%, 70.5%, and 62.5% in the first three audits, respectively). These findings highlight both improvements in prescription accuracy and dietitian review, and ongoing areas requiring quality improvement, particularly nutritional monitoring.

Overall, this re-audit demonstrates meaningful progress in nutritional care, particularly through improved dietitian involvement, correct TPN prescription, and consistent documentation of TPN indications. However, the decline in BMI and weight recording highlights a gap in basic clinical documentation that requires attention. While other aspects of TPN management remained stable, these findings emphasise the need to maintain both the quality of clinical practice and the accuracy of documentation through ongoing education and regular audit.

Limitations

Our audit has a few limitations. The sample size was small and included only surgical patients receiving TPN outside intensive/critical care, which may limit the generalizability of the findings. Data were collected retrospectively, which posed some difficulty in locating and obtaining the required information due to poor documentation. Although a statistically significant difference was observed in correct TPN prescription and dietitian review, the audit was not powered to detect smaller changes in other variables, such as OOH doctors-mediated initiation of TPN.

Despite these limitations, the audit provides valuable insights into the practice of TPN, highlights persistent gaps in documentation, and identifies targeted areas for quality improvement, particularly in nutrition monitoring and timely involvement of dietitians.

## Conclusions

The findings of the audit across four cycles demonstrate that our centre has made meaningful progression to adherence with local and national standards for the delivery of parenteral nutrition, particularly with regard to accurate prescription and dietitian-guided initiation. The continued high use of OOH "Emergency TPN" remains an area requiring focused intervention and aligns with concerns highlighted in the report of NCEPOD.

The evidence suggests that a multidisciplinary team involvement, including dietitians/clinical nutritionists, physicians/surgeons, pharmacists, and nurses, is central to ensuring high-quality standards in the delivery of TPN. Improved education, clearer documentation pathways, and strengthened communication may support a more consistent and streamlined practice. Regular audit and feedback cycles of our practice are of paramount importance for improving patient care in complex areas of healthcare delivery, such as TPN. Although the findings of this audit may relate to local practices, the lessons learnt may be informative for other healthcare centres aiming to evaluate their TPN governance and quality improvement processes. Overall, this audit provides a practical framework for monitoring, improving, and sustaining high standards of TPN delivery within surgical services, in line with clinical governance principles.
